# New Aspects of Secretory Structures in Five Alismataceae Species: Laticifers or Ducts?

**DOI:** 10.3390/plants10122694

**Published:** 2021-12-08

**Authors:** Flávia Maria Leme, João Pedro Silvério Pena Bento, Vitoria Silva Fabiano, Jean David Varilla González, Vali Joana Pott, Rosani do Carmo de Oliveira Arruda

**Affiliations:** 1 Laboratório de Anatomia Vegetal, Instituto de Biociências, Universidade Federal de Mato Grosso do Sul, Cidade Universitária, Caixa Postal 549, Campo Grande 79070-900, MS, Brazil; joaospena@gmail.com (J.P.S.P.B.); vitoria.fabiano@ufms.br (V.S.F.); jvarillabiologia@gmail.com (J.D.V.G.); 2 Programa de Pós-Graduação em Biologia Vegetal, Instituto de Biociências, Universidade Federal de Mato Grosso do Sul, Cidade Universitária, Caixa Postal 549, Campo Grande 79070-900, MS, Brazil; 3 Herbário CGMS, Instituto de Biociências, Universidade Federal de Mato Grosso do Sul, Cidade Universitária, Caixa Postal 549, Campo Grande 79070-900, MS, Brazil; vali.pott@gmail.com

**Keywords:** *Echinodorus*, *Helanthium*, *Hydrocleys*, *Limnocharis*, ontogenesis, *Sagittaria*, secretory ducts

## Abstract

The secretory structures of Alismataceae have been described as secretory ducts, laticifer ducts, laticifer canals or schizogenous ducts. However, these terms are not found in the specialized literature, and ontogenetic analyses for the exact classification of these structures are missing. Accordingly, more studies regarding the secretory structures of Alismataceae are necessary to establish homology in the family or in the order. Thus, the aim of this study was to describe the anatomy, ontogeny, distribution in the organs and exudate composition of the secretory structures present in five Alismataceae species in order to determine whether the family has laticifers or secretory ducts. Samples of leaves, flowers and floral apices were processed for anatomical and histochemical analyses by light microscopy. The analysis indicated the presence of anastomosing secretory ducts in all species, occurring in both leaves and flowers. The exudate contains lipids, alkaloids, proteins and polysaccharides, including mucilage. The secretory duct structure, distribution and exudate composition suggest a defense role against herbivory and in wound sealing. The presence of secretory ducts in all species analyzed indicates a probable synapomorphy for the family.

## 1. Introduction

Alismataceae is a family consisting of floating to emergent aquatic or marsh herbs with a worldwide distribution [1]. Limnocharitaceae was merged with Alismataceae to form one family comprising 17 genera [1,2,3,4] and ca. 100 species [5]. The family is known for including species with economic uses as food sources [6], ornamentals (aquarium plants) [6,7] and medicinals [6,7], as well as species for beekeeping [1,6]. The largest genera are *Echinodorus* and *Sagittaria,* both regularly used as aquarium and pond ornamental plants [1,6]. Leaves of *Echinodorus grandiflorus* (Cham. and Schltdl.) Micheli and *E. macrophyllus* (Kunth) Micheli are used for medicinal purposes [7,8,9,10,11,12]. Ethnobotanical investigations and clinical trials indicate that *E. grandiflorus* leaves have anti-hypertensive, anti-inflammatory, diuretic, and anti-arthritic properties [7,10,11,12,13].

Laticifers have been reported in some families of Alismatales [14], such as Aponogetonaceae [15], Araceae [16,17] and Alismataceae [14]. According to other authors, laticifers have not been recorded in Alismataceae [18,19] and Butomaceae [20], while resin secretory ducts have been described only for Araceae [14].

The secretory structures of Alismataceae have been described as secretory ducts, laticifer ducts or laticifer canals [1,19,20,21,22,23,24], and the secretion is described as latex [23] or milky juice [1]. However, laticifer ducts or laticifer canals are not found in the specialized literature on secretory structures [25,26,27]. In addition, the lack of ontogenetic analyses for the exact classification of these structures does not allow the establishment of homology in the family or order [23].

The secretory ducts or canals are elongated secretory structures lined with an epithelium of live secretory cells delimiting a large intercellular space (lumen) [14,25,26,27,28]. Ontogenetically, they may develop by schizogeny (separation of cells), lysigeny (disintegration of cells) or both (separation and disintegration of cells) [25]. Their development occurs from a group of a few initial meristematic cells that form a rosette in cross-section. These cells undergo divisions in various directions, and the rosette becomes more distinct from the surrounding cells, constituting the future epithelium [14,25,27,29]. The lumen develops in the middle of the epithelium, and further periclinal divisions may occur outside the epithelium, forming a sheath with one or more cell layers. The material secreted by the duct varies between resin, gum or mucilage [14,25,26,29].

Laticifers are a specialized type of secretory structure with an emulsion or suspension of compounds of a varied nature, in which terpenoids predominate, called “latex” [25,26,27,28,30,31]. The latex color may vary according to composition; it may be white (milky) [32,33], yellow [30,34], orange [35], red [36] or colorless [34]. Laticifers consist of one cell with intrusive growth (nonarticulated non-anastomosing type) or a series of connected cells (articulated anastomosing type) that form a uniseriate tube [25,30,34,37]. Articulated anastomosing laticifers or nonarticulated laticifers can be branched or unbranched [25,26,27,30,34,37]. Articulated anastomosing laticifers in a mature phase have terminal walls that disintegrate (multinucleated structure), that can branch and can assume several forms [25,30,34,37].

Laticifers and secretory ducts have been cited in at least 40 and 50 families, respectively, of vascular plants, including phylogenetically unrelated plants such as ferns, gymnosperms, and angiosperms, and they have emerged many times in the course of plant evolution [14,30,38]. Laticifers and secretory ducts have roles in herbivory reduction or resistance in plants. Their products (latex–laticifers; resin, gum or mucilage–secretory ducts) are of great economic importance since they are crucial for the production of pharmaceuticals, enzymes and rubber [28,31,39].

Although there are several anatomical studies on Alismataceae species [19] such as *Echinodorus* spp. [23,24], *Helanthium tenellum* [7], *Alisma plantago* [40], *Echinodorus macrophyllus* [41] and *Sagittaria montevidensis* [22], these studies do not provide details regarding the anatomy and ontogeny of the secretory structures called the “laticifer ducts” [1,11,14,22,23,24] or secretory canals [41]. Histochemical studies on the composition of the secretion are also absent. Thus, there is nothing to indicate whether the secreted material has latex or resin characteristics. Therefore, our objective was to study the structure, ontogeny, distribution in the organs and secretion composition of the secretory structures present in *Echinodorus grandiflorus, H. tenellum*, *Hydrocleys nymphoides, Limnocharis flava* and *Sagittaria rhombifolia* in order to determine whether the analyzed species have laticifers or secretory ducts.

## 2. Results

**Figure 1:**
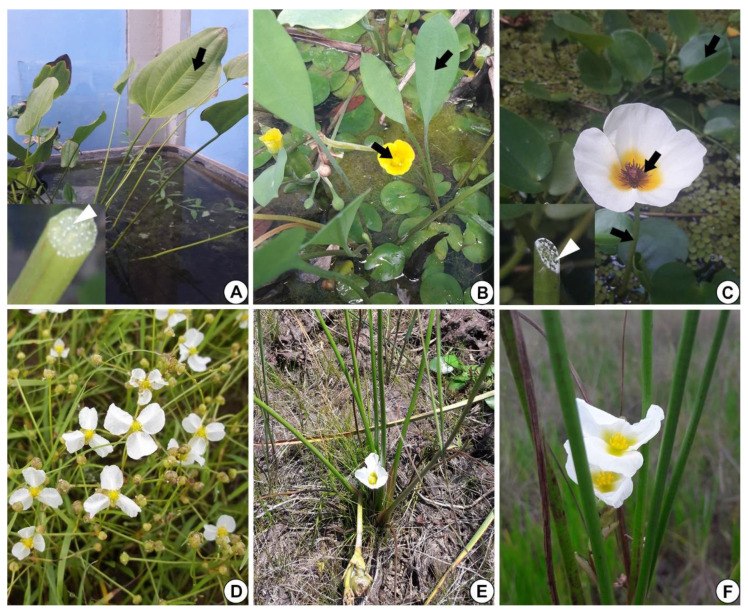
Alismataceae species analyzed. (**A**) *Echinodorus grandiflorus* leaf (arrow). Inset: detail of the petiole with white secretion (white arrowhead). (**B**) *Limnocharis flava* showing leaves and flowers (arrows). (**C**) *Hydrocleys nymphoides*: leaf, petiole and flower (arrows). Inset: note on the left side the petiole with white secretion (white arrowhead). (**D**) *Helanthium tenellum* (Image **D**: Giseli Catian). (**E**,**F**) *Sagittaria rhombifolia*.

**Figure 2:**
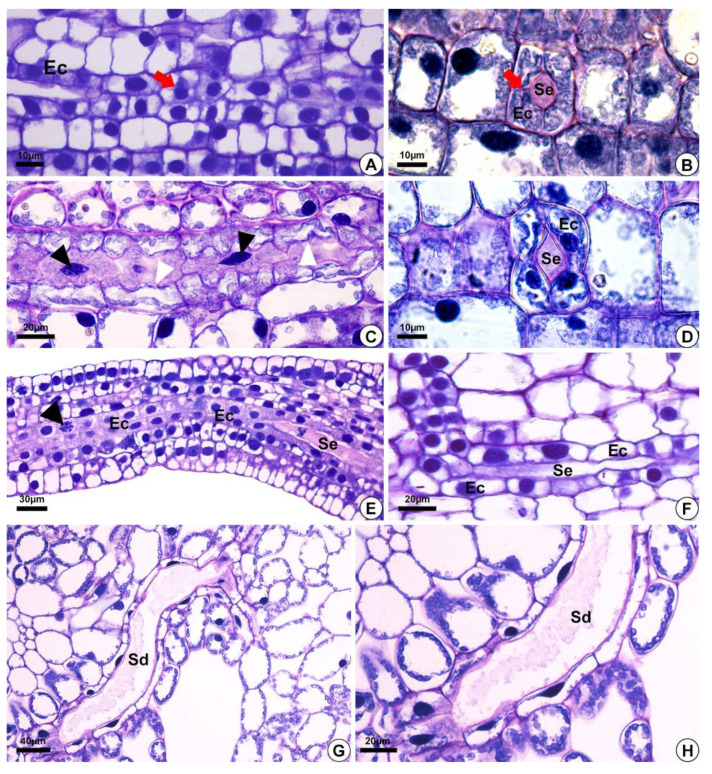
Light micrographs after staining with toluidine blue, depicting the development of secretory ducts in *Helanthium tenellum*, *Hydrocleys nymphoides, Limnocharis flava* and *Sagittaria rhombifolia*. (**A**,**F**) *Hydrocleys nymphoides*. (**B**–**D**) *Limnocharis flava*. (**E**) *Helanthium tenellum*. (**G**,**H**) *Sagittaria rhombifolia*. Longitudinal (**A**,**C**,**E**,**F**) and cross-section (**B**,**D**) of the floral meristem. (**A**,**B**) Secretory ducts originated from the ground meristem by asymmetrical mitotic divisions (red arrow) (**A**). The initial epithelial cells start to pull away (schizogeny) and give rise to the lumen (**B**). (**C**) Secretory duct development. Note epithelial cells with fusiform nuclei (black arrowheads), and an undulating cell wall (white arrowheads). (**D**–**F**) Epithelial cells with large nuclei and dense cytoplasm. (**E**) Epithelial cells in division (arrowhead), with an increase in the number of secretory cells around the lumen. (**G**,**H**) Mature secretory ducts. (**G**) Branching of secretory ducts by anastomosis. (**H**) Note the presence of secretion. Sd = secretory ducts. Ec = epithelial cells. Se = secretion.

**Figure 3:**
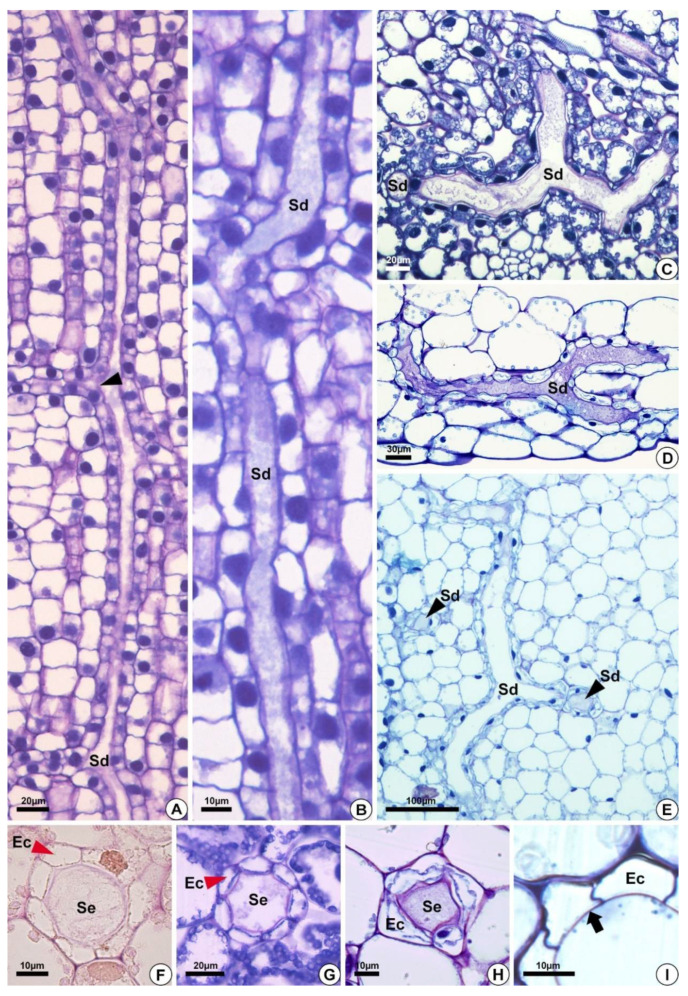
Structure of the secretory ducts of *Hydrocleys nymphoides* (**A**,**B**), *Sagittaria rhombifolia* (**C**,**F**,**G**), *Limnocharis flava* (**D**,**E**,**H**), and *Echinodorus grandiflorus* (**I**). (**A**–**E**) Longitudinal sections. (**F**–**I**) Cross-sections showing secretory ducts in the aerenchyma tissue of the petiole. All preparations were stained with toluidine blue, except for the one shown in (**F**), which was stained with Schiff reagent (PAS). (**A**) Secretory ducts with anastomoses in different directions (black arrowhead). (**B**) Elongated secretory ducts. (**C**–**E**) Anastomosing secretory ducts “Y”-shaped. (**D**) Epithelial cells surrounding the lumen. (**E**) Secretory ducts in longitudinal (Sd) and cross-sections (black arrowheads–Sd). (**F**–**H**) Cross-section showing secretory ducts with a layer of epithelium (red arrowheads) composed of variable epithelial cell numbers. (**I**) Pectocellulosic cell wall of epithelial cells (arrows). Sd = secretory ducts. Ec = epithelial cells. Se = secretion.

In *E. grandiflorus, H. tenellum*, *H. nymphoides, L. flava* and *S. rhombifolia* (Figure 1A–F)*,* we found elongated secretory ducts consisting of an intercellular space or lumen lined with one layer of secretory cells, i.e., the epithelium. The lumen, where the secretion is released and stored, is formed by schizogeny during organ development (Figure 2A–F and Figure 3A–H). In the species evaluated, the secretory ducts are extremely narrow, with a diameter ranging from 11.9 µm in *H. tenellum* to 41.8 µm in *H. nymphoides*.

### 2.1. Origin and Morphology of the Secretory Structures

The secretory ducts of *E. grandiflorus*, *H. tenellum*, *H. nymphoides*, *L. flava* and *S. rhombifolia* differentiate early during organ development, being fully visible while other tissues are still meristematic (Figure 2A–E). Thus, the secretory ducts are formed before the complete development of the surrounding tissues. Secretory ducts originate from the ground meristem (Figure 2A–E) by asymmetrical mitotic divisions, followed by the dissolution of the middle lamella between the cells, which gives rise to the lumen in the early stage of development when the epithelium is composed of only four cells (Figure 2B–D). Initially, the cells of the rosette have large nuclei and a dense and uniform cytoplasm. These initial cells are distinguished from the adjacent meristematic cells by asymmetrical cell divisions (Figure 2A,B), dense cytoplasm and fusiform nuclei (Figure 2C). The wall of the epithelial cells becomes progressively undulating in *L. flava* (Figure 2C); however, in *S. rhombifolia,* the cell wall is smooth throughout development (Figure 2G,H). The ducts branch through lateral anastomoses with other secretory ducts (Figure 2G). At maturity, most ducts form an interconnected network of canals that extend longitudinally and radially throughout the shoot system (Figure 3A–E). Branched ducts are easily recognized by specific shapes such as a Y-bifurcation pattern (Figure 3C–E).

All species analyzed have ducts with a single-layered epithelium (Figure 3F–H). However, the epithelial cells are morphologically variable in length and width. They are large in *H. tenellum* and *H. nymphoides* (Figure 3A,B) but small and narrow in *E. grandiflorus, S. rhombifolia* and *L. flava* (Figure 3C–E). In mature secretory ducts, the epithelium is composed of 5 to 8 cells (Figure 3F–H) as seen in cross-section in all species analyzed. Epithelial cells generally have a flattened shape and thin cell walls that project into the lumen, have a slightly dense cytoplasm and large nuclei and contain small plastids. (Figure 3). The cell wall is pectocellulosic, reacting positively with Schiff’s reagent (PAS) (Figure 3F) and staining magenta with toluidine blue (Figure 3H,I).

### 2.2. Distribution of the Secretory Ducts in the Plant

**Figure 4:**
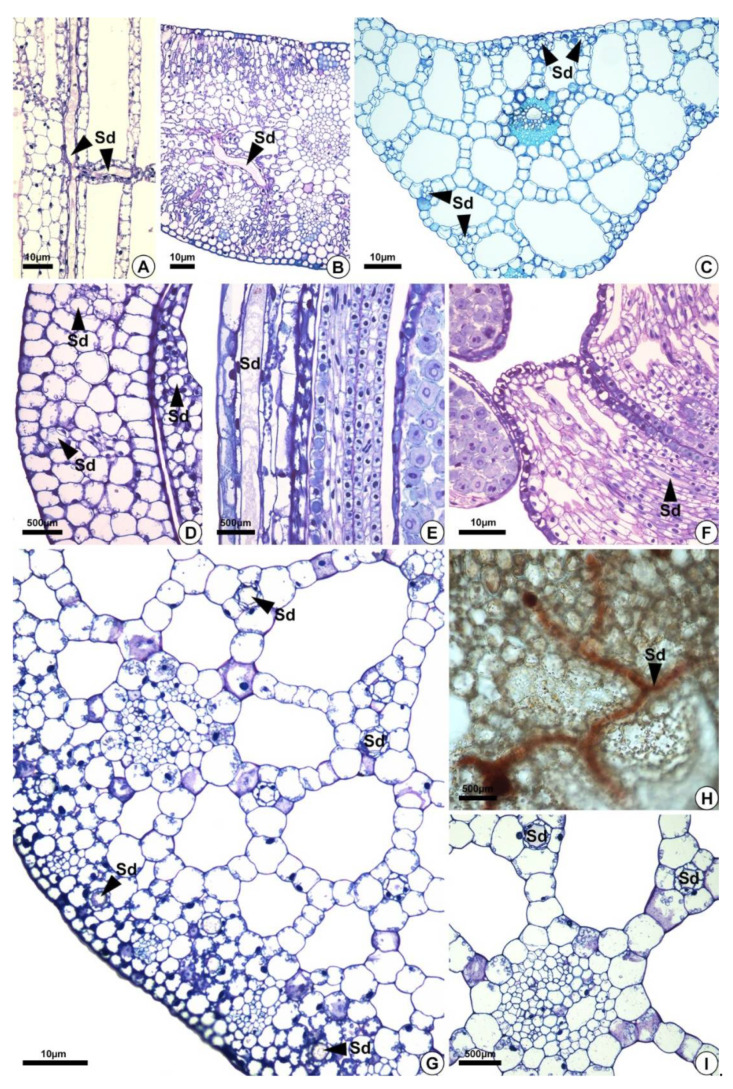
Distribution of secretory ducts in the petiole, leaf blade, and perianth of *Sagittaria rhombifolia* and *Helanthium tenellum*. (**A**,**B**,**D**–**I**) *Sagittaria rhombifolia*. (**C**) *Helanthium tenellum.* All preparations were stained with toluidine blue, except for (**H**) which was stained with oil red reagent that reacted positively in orange for lipids. (**A**) Longitudinal section of the petiole. Note the secretory ducts distributed vertically and longitudinally in the aerenchyma (arrowheads). (**B**,**C**) Secretory ducts in the leaf blade and the midrib of a cross-section. (**D**–**F**) Longitudinal section of the floral organs. (**D**,**E**) Secretory ducts in the perianth (arrowheads). (**F**) Secretory ducts in the filament (arrowheads). (**G**–**I**) Cross-sections. (**G**) Cross-section of a petiole. Note the secretory ducts in the cortex and the aerenchyma (arrowheads). (**H**) Secretory ducts in the diaphragm showing lipids in orange. (**I**) Secretory ducts in the aerenchyma around the vascular bundle. Sd = secretory ducts.

Secretory ducts are present in the leaves (petiole, blade and midrib) of the five species analyzed and in the flowers of *H. nymphoides, L. flava,* and *S. rhombifolia* (Table 1, Figure 4). They occur at higher frequency in *H. nymphoides, L. flava*, and *S. rhombifolia* and at lower frequency in *H. tenellum* and *E. grandiflorus*. In the five species studied, secretory ducts were found in the petiole (Figure 4A), leaf blade, and midrib (Figure 4B,C). In *H. nymphoides, L. flava,* and *S. rhombifolia* flowers, the secretory ducts were distributed in the perianth and stamens (Figure 4D–F). The secretory ducts occur in the subepidermal layer, mesophyll, aerenchyma tissues and diaphragm (Figure 4). In the aerenchyma, they are distributed around the vascular bundles and pass among the aerenchyma spaces (Figure 4G–I).

### 2.3. Secretion Composition

**Figure 5:**
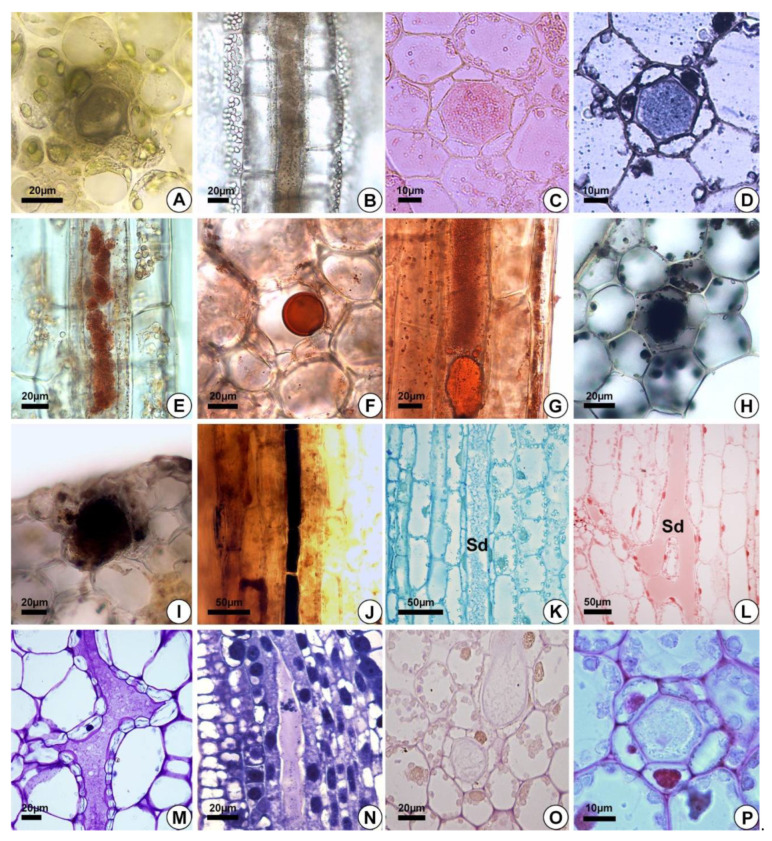
Histochemical analyses of the secretion. (**A**,**F**,**G**,**L**,**P**) *Hydrocleys nymphoides*. (**B**–**E**,**K**,**O**) *Sagittaria rhombifolia*. (**J**,**N**) *Helanthium tenellum.* (**I**,**M**) *Limnocharis flava*. (**H**) *Echinodorus grandiflorus*. (**A**,**B**) Secretory ducts without staining. (**C**–**G**) Positive reaction of the secretion for total lipids (Sudan IV (**C**), Sudan black (**D**) and oil red (**E**–**G**)). (**H**–**J**) Alkaloids (Wagner’s reagent). (**K**,**L**) Proteins (Coomassie blue (**K**) and xylidine Ponceau (**L**)). (**M**,**N**) Secretion with acidic substances (toluidine blue). (**O**) Neutral polysaccharides (PAS). (**P**) Positive reaction of the secretion for acidic mucilage (Ruthenium red). Sd = secretory ducts.

The secretion in the leaves and petioles of *E. grandiflorus*, *H. tenellum, H. nymphoides, L. flava* and *S. rhombifolia* was milky (Figure 1A,C). Compared to the other species analyzed, *H. nymphoides* showed the greatest exudation of secretion. The secretion was initially fluid and became thicker after air contact. The histochemical tests were performed on the petiole of the leaves (Figure 5A–K,P) or on floral buds (Figure 5L–O). The results demonstrated that the secretion produced by the secretory ducts was of resin and consisted mainly of lipids (Figure 5C–G), alkaloids (Figure 5H–J), proteins (Figure 5K,L) and polysaccharides (Figure 5M–O) (Table 2). The analysis showed that the secretion was white in fresh material (Figure 1A,C) and dark on the slide (Figure 5A,B). For lipids, the Sudan and the oil red tests were positive in all species of Alismataceae analyzed (Figure 5E–G). We also detected alkaloids (Figure 5H–J), proteins (Figure 5K,L), and polysaccharides (Figure 5M–P) including mucilage (Figure 5M,N,P) in the secretory ducts. Polysaccharides were weakly stained (Figure 5O). No phenolic compounds were found (Table 2).

## 3. Discussion

Our data support the presence of secretory ducts in *E. grandiflorus, H. tenellum*, *H. nymphoides, L. flava*, and *S. rhombifolia*, as evidenced by anatomy, ontogeny and exudate composition analysis. The presence of resin ducts (*sensu lato*) in five genera of Alismataceae is a novelty for the family since controversial terms for these secretory structures were reported in previous studies, and secretion was reported as latex [1,14,19,22,23,24,41]. The development of resin ducts begins very early during plant development, differentiating from meristematic tissues in the reproductive and vegetative apices. We observed that resin ducts initiate with asymmetrical cell division, forming four epithelial cells surrounding a lumen. These cells divide again, adding new cells to the epithelium. During development, the secretory ducts anastomose laterally, forming an interconnected network of canals which store the resin composed of varied substances, as typically described for the exudate of some secretory ducts in angiosperms [14,25]. Therefore, our results corroborate the presence of secretory ducts [1,19,22,41] and refute the use of the terms laticifer [14] or laticiferous duct [23,24,42] in *E. grandiflorus, H. tenellum, H. nymphoides, L. flava* and *S. rhombifolia*. The confusion of terms used by researchers is due to the difficulty in identifying the real secretory structure present in the members of Alismataceae. Features such as the reduced diameter of the secretory duct, heterogeneous secretion composition, the lack of a sheath surrounding the epithelium, and the white color of the exudate likely caused this misinterpretation. This fact has also occurred in Anacardiaceae, Burseraceae, Clusiaceae, Cactaceae and Calophyllaceae [14,29,43].

### 3.1. Resin Duct Structure

The anatomy of the internal secretory structures observed in *E. grandiflorus, H. tenellum, H. nymphoides, L. flava* and *S. rhombifolia* is consistent with the secretory duct definition: a structure formed by an epithelium of secretory cells that delimits an intercellular space (lumen), where the secretion is stored. In Alismataceae, the ducts produce a complex resin which contains terpenes, phenolic compounds, proteins and polysaccharides, as observed in other families [14,25,26,27]. Notably, the resin ducts of Alismataceae are narrow and lack a sheath (this study), as observed in the ducts of Clusiaceae [29,44]. Sheaths formed by two cell layers surrounding the duct occur in Anacardiaceae [45], Hypericaceae [46], and Araceae [47]. Narrow secretory ducts are not common in angiosperms, but they have also been reported in *Philodendron adamantinum* Mart. ex Schott (Araceae) [47] and *Hypericum perforatum* L. (Hypericaceae) [46].

In the present study, epithelial cells have thin walls [44,48], dense cytoplasm mainly during the secretory phase, a fact also observed in *Garcinia mangostana* L. (Clusiaceae) [49] and *Clusia* species [29,44], in addition to large vacuoles and many mitochondria, plastids and Golgi apparatus. For Araceae, two types of secretory ducts have been described: one of small diameter in which the secretion accumulates in its lumen and is related to plant defense, with release of the secretion occurring only in the case of injury, and the other of large diameter that releases resin into the external environment and is directly related to the pollination mechanism, having the function to guarantee pollen adherence, specifically present in the adaxial part of the spathe of the inflorescence [47]. The first one is the most common in species of the family and is distributed in roots, stem, leaves and floral organs [47,50]. Although more details are needed to compare Alismataceae ducts to Araceae secretory ducts “type I” [47,50], the structure and diameter show similarities that might be of phylogenetic origin.

The ontogeny of the secretory duct was characterized by asymmetrical mitotic divisions of meristematic cells, followed by the dissolution of the middle lamella between them, resulting in lumen expansion (this study). In Alismataceae species, the lumen is formed in the early stage of duct ontogeny, with secretion already present, and the epithelium has only four cells. In other species, e.g., Clusiaceae, the initial rosette has various cells that will then form the lumen [29,44].

### 3.2. Secretion Composition and Functions

The resin detected here has a complex composition, containing different chemical classes of substances. Most components are lipophilic, but others have been identified, such as alkaloids, proteins and polysaccharides, including mucilage. Generally, the secretion of secretory ducts may be classified as resin, mucilage or gum [14,25] according to its composition. Mucilage ducts occur in Malvaceae [51] and Calophyllaceae [38]; resin ducts in Araceae [47], Clusiaceae [29] and Anacardiaceae [45]; and gum ducts in Calophyllaceae [38]. However, the complex composition of some resins, which confers white color to the exudate, has caused misinterpretation and has led some researchers to call it “latex”, as in the case of Alismataceae ([19,20], this study), Anacardiaceae [14,52,53] and Clusiaceae [14,44]. Nevertheless, latex is produced only by laticifers [14,25].

The resin produced by ducts is usually related to plant defense against herbivores [28,54,55,56,57]. The presence of terpenes in the resin of Alismataceae indicates that it can provide wound sealing. When the plant is damaged, resin overflows and the terpenes arapidly coagulate when in contact with the air, thus sealing the wounds [55,58]. An anti-herbivory action may also occur because the resin can trap whole insects or their mouthparts [55].

The distribution of resin ducts in the cortex, aerenchyma and surrounding vascular bundles confirms the important defensive and wound healing role of these structures, which are abundantly distributed from peripheral to internal tissues [59,60,61] in *E. grandiflorus, H. tenellum, H. nymphoides, L. flava* and *S. rhombifolia*.

### 3.3. Taxonomic Implications

The families that make up the order Alismatales are characterized by different internal secretory structures such as secretory ducts (resin ducts), laticifers and tanniniferous idioblasts [1,14,19,62]. Secretory ducts are present only in Alismataceae and Araceae, while laticifers are present in Aponogetonaceae, Araceae and Juncaginaceae and tanniniferous idioblasts occur in many families of the order (see Table 3). Of the 14 Alismatales families, only two have secretory ducts and are not closely related according to molecular analysis [63]. Thus, the secretory structures demonstrate an independent origin in the order, as observed in other orders such as Sapindales [14,45].

The presence of secretory ducts in Limnocharitaceae and Alismataceae had led some researchers to suggest that Limnocharitaceae should be included in Alismataceae [18], as later confirmed by molecular analysis [2].

Secretory ducts have been reported for 11 out of 17 genera of Alismataceae [19, this study]. In other genera such as *Luronium,* the secretory duct was not observed in the Stant study [19], and in the genera *Astonia, Albidella, Caldesia, Butomopsis* and *Burnatia* it has not been histologically studied so far. Therefore, the presence of secretory ducts appears to be a putative synapomorphy of Alismataceae. Here, we suggest checking their absence in *Luronium* [19] by electron microscopy, which can favor a better identification of the secretory structure. Anatomical studies of the other five genera are also essential in order to confirm the synapomorphy of the family.

In taxonomic approaches, Alismataceae secretory ducts are called “puncta pellucida” and “lineae pellucidae” common in *Echinodorus*, an important diagnostic feature for some species [18] and are apparently the regions where secretory ducts are next to or in contact with the epidermis. Accordingly, the presence and/or distribution of secretory ducts may be an important taxonomic characteristic for species of Alismataceae at the genus and species levels. Therefore, anatomical studies may be of help in the understanding of this plant group and in the elucidation of the convergent relationships between plants and insects from an ecological point of view.

## 4. Materials and Methods

### 4.1. Plant Material

Samples of *Echinodorus grandiflorus* (Cham. and Schltr.) Micheli (43064 CGMS)*, Helanthium tenellum* (Mart. ex Schult. and Schult. f.) Britton (V.J. Pott—n° 8420), *Hydrocleys nymphoides* (Willd.) Buchenau (V.J. Pott—n°6395)*, Limnocharis flava* (L) Buchenau (V.J. Pott—n° 2561), and *Sagittaria rhombifolia* Cham. (V.J. Pott—n° 4272), were collected in the aquatic plant aquarium of the Laboratório de Botânica of Instituto de Biociências, Universidade Federal de Mato Grosso do Sul (INBIO/UFMS) and photographed with a cell phone camera of 8 Megapixel (Samsung, Daegu, Korea) (Figure 1). Vouchers were deposited in the CGMS herbarium (INBIO/UFMS). Leaf samples of all species were studied. For *H. nymphoides, L. flava* and *S. rhombifolia,* floral scapes and flowers were also analyzed. In addition, floral apices were analyzed for *L. flava* and *S. rhombifolia.*

### 4.2. Histological Analysis

For the histological study (light microscopy—LM), the materials collected were fixed in buffered formalin for 48 h to preserve lipophilic and phenolic substances [66], or in formalin–acetic acid–ethanol (50% FAA) for 48 h to preserve hydrophilic substances [67]. Leaves, flowers and floral apices were dehydrated with an ethanol series (10%, 30%, 50%, 70%, 80%, 90% and 95%), embedded in histological resin using a preinfiltration solution (95% alcohol: pure resin, 1:1) and a pure resin solution (Historesin, Leica Microsystems Inc., Heidelberg, Germany), and cut into longitudinal and transverse 5 µm sections using a rotary microtome (Leica RM 2145, Leica Microsystems Inc., Heidelberg, Germany). The sections were stained with 0.1% toluidine blue in phosphate buffer, pH 6.5 [68,69,70,71], washed under running water, dried in the open air, mounted in Entellan^®^ (Merck KGaA, Darmstadt, Germany) and observed under a Nikon Eclipse Ci light microscope (Tokyo, Japan) with a Motic^®^ Cam Pro 252B digital camera (Beijing, China).

### 4.3. Histochemical Analysis

Petiole samples were also free-hand sectioned using fresh material, with five fragments and more than 20 sections for each, and the main compounds of the exudate were analyzed using the following reagents: Sudan Black B and Sudan IV for total lipids [72], oil red for rubber [73], Lugol for starch [66,73], ferric chloride for phenolic compounds [66], and Wagner’s reagent for alkaloids [73,74]. Material embedded in historesin was stained with toluidine blue for the detection of phenolic compounds and pectin [67], with periodic acid-Schiff (PAS) for neutral polysaccharides [73,75] and Ruthenium red for acidic mucilage [76], and with Coomassie blue [73,77] and xylidine Ponceau [73,78] for proteins (see [68,70,71]). Photomicrographs were obtained with a Leica DFC 495 digital camera coupled to a Leica DM 5500 B light microscope and a Nikon Eclipse Ci photomicroscope (Tokyo, Japan) with a Motic^®^ (Beijing, China) Cam Pro 252B digital camera.

## 5. Conclusions

This research demonstrated and confirmed the presence of resin ducts in *Echinodorus grandiflorus, Helanthium tenellum*, *Hydrocleys nymphoides, Limnocharis flava* and *Sagittaria rhombifolia*. The resin ducts are formed by separation of cells (schizogeny) and the epithelium has cells with thin walls, a dense cytoplasm and large nuclei. The ducts are abundantly distributed close to the epidermis and in the aerenchyma. Lipids, proteins and alkaloids were detected in the secretion. The structure, distribution and secretion composition of the secretory ducts suggest their defensive role against herbivory and a protective function in wound sealing. Our novel data reveal a gap of knowledge about ducts in Alismataceae, with further in-depth analysis, including ultrastructural and chemical investigation, being needed to better understand the secretion mechanism of this secretory structure.

## Figures and Tables

**Table 1:** Distribution of secretory ducts in the vegetative and floral organs of *Echinodorus grandiflorus*, *Helanthium tenellum, Hydrocleys nymphoides, Limnocharis flava* and *Sagittaria rhombifolia*. Symbols: (+) presence; (−) absence; (NA) not analyzed.

	Organ	*Echinodorus grandiflorus*	*Helanthium tenellum*	*Hydrocleys nymphoides*	*Limnocharis flava*	*Sagittaria rhombifolia*
Leaf	Petiole	+	+	+	+	+
	Blade	+	+	+	+	+
Flower	Floral scape	NA	NA	+	+	+
	Floral organs	NA	NA	+	+	+

**Table 2:** Histochemical data obtained for the secretory duct secretion of *Echinodorus grandiflorus*, *Helanthium tenellum, Hydrocleys nymphoides, Limnocharis flava* and *Sagittaria rhombifolia*. Symbols: (+) presence; (−) absence.

Reagent	Target Compound	Color	*Echinodorus grandiflorus*	*Helanthium tenellum*	*Hydrocleys nymphoides*	*Limnocharis flava*	*Sagittaria rhombifolia*
Schiff (PAS)	Neutral polysaccharides	pink	+	−	+	+	+
Lugol	Starch grains	blue	−	−	−	−	−
Sudan Black B	Total lipids	black	+	+	+	+	+
Sudan IV	Total lipids	light orange	+	+	+	+	+
Oil Red	Lipids	orange	+	+	+	+	+
Comassie Blue	Protein	blue	+	+	+	+	+
Wagner’s reagent	Alkaloids	blue-black to reddish	+	+	+	+	+
Toluidine Blue	Phenolic compounds	green	−	−	−	−	−
Ferric chloride	Phenolic compounds	brownish	−	−	−	−	−

**Table 3:** Survey of secretory structures in Alismatales based on the specialized literature.

Family/Species	Laticifers	Secretory Canal	Secretory Structure	References
Alismataceae *Echinodorus grandiflorus, Helanthium tenellum, Hydrocleys nymphoides, Limnocharis flava, Sagittaria rhombifolia*	Absent	Present	Secretory ducts anastomosed	This study
Alismataceae	Present	Absent	Not specified	[14]
Alismataceae (Limnocharitaceae)	Not specified	Not specified	Secretory ducts (“laticifers”)	[1,18,19]
Alismataceae	Not specified	Not specified	Secretory ducts or latex canal	[42]
*Echinodorus macrophyllus*	Not specified	Present	Secretory canal	[41]
*E. glandulosus, E. lanceolatus, E. palaefolius, E. paniculatus, E. pubescens, E. subalatus* subsp. *subalatus.*	Not specified	Not specified	Laticiferous ducts	[24]
*Sagittaria guayanensis* ssp. *lappula*	Not specified	Not specified	Laticifers or laticifer canal (articulated)	[11]
*Sagittaria montevidensis*	Not specified	Not specified	Schizogenous duct	[22]
*Sagittaria acutifolia, Alisma plantago, Baldellia ranunculoides, Damasonium alisma, Echinodorus, Liminophyton obtusifolium, Ranalisma humile, Wisneria schweinfurthii*	Not specified	Not specified	Secretory ducts	[19]
*Sagittarialatifolia, Luronium natans* and *Wisneria*	Not specified	Not specified	Tannin cells	[19]
Aponogetonaceae	Present	Absent		[14]
	Present	Absent		[15]
	Present	Absent	Articulated laticifers and tannin cells	[64]
Araceae	Present	Present	Not specified	[14,17]
	Present	Present	Not specified	[16,50]
Butomaceae	Absent	Absent	Not specified	[18]
Cymodoceaceae	Absent	Absent	Tannin cells	[18]
Hydrocharitaceae	Absent	Absent	Absence of schizogenous secretory ducts	[19,65]
Juncaginaceae *Triglochin*	Present	Absent	Not specified	[1,20]
Maundiaceae	Absent	Absent	Not specified	[1]
Posidoniaceae	Absent	Absent	Tannin cells	[62]
Ruppiaceae	Absent	Absent	Tannin cells	[1]
Scheuchzeriaceae	Absent	Absent	Tannin cells	[18]

## Data Availability

Data is contained within the article.
